# Efficacy and functionality of sugarcane original vinegar on mice

**DOI:** 10.3389/fmicb.2023.1224666

**Published:** 2023-08-07

**Authors:** Feng-Jin Zheng, Bo Lin, Yu-Xia Yang, Xiao-Chun Fang, Krishan K. Verma, Gan-Lin Chen

**Affiliations:** ^1^Institute of Agro-Products Processing Science and Technology, Guangxi Academy of Agricultural Sciences, Nanning, Guangxi, China; ^2^Guangxi Key Laboratory of Fruits and Vegetables Storage-Processing Technology, Nanning, China; ^3^Key Laboratory of Sugarcane Biotechnology and Genetic Improvement (Guangxi), Ministry of Agriculture and Rural Affairs, Guangxi Key Laboratory of Sugarcane Genetic Improvement, Sugarcane Research Institute, Guangxi Academy of Agricultural Sciences, Nanning, Guangxi, China; ^4^School of Chemistry and Chemical Engineering, Guangxi Minzu University, Nanning, Guangxi, China

**Keywords:** sugarcane original vinegar, safety, functionality, evaluation, mice

## Abstract

**Introduction:**

Due to their bioactive compounds and beneficial health effects, functional foods and plant-based natural medicines are widely consumed. Due to its bioactivities, vinegar is one of them that helps humans. Sugarcane original vinegar (SOV) is a special vinegar made from sugarcane as a raw material through biological fermentation processes.

**Methods:**

The objective of this study was to assess the effects of sugarcane original vinegar on growth performance, immune response, acute oral toxicity, bacterial reverse mutation, mammalian erythrocyte micronucleus, mouse spermatogonial chromosome aberration, mammalian bone marrow cell chromosome aberration changes, and serum characteristics in mice. Distortion parameters were used to assess its safety, and at the same time, the functionality of SOV was monitored during experimentation.

**Results:**

The results show that the SOV has no damage or inhibitory effect on the bone marrow red blood cells of mice and no mutagenic or distortion-inducing effects on the bone marrow cell chromosomes or spermatogonia chromosomes, so it is safe to eat. SOV can improve blood lipids and reduce blood lipid content.

**Discussion:**

The study results provide data basis for the intensive processing of sugarcane and the development of high-value SOV products. Sugarcane original vinegar has a beneficial impact on performance, immune response, and chromosomal aberration. The production application influences the vinegar's quality and, consequently, its health benefits.

## Introduction

Sugarcane vinegar is sweet and sour brewed from sugar crops, i.e., sugarcane, which is prepared by extracting juice from sugarcane mature stems, sterilizing them, and then undergoing alcoholic (Zheng et al., 2018; Luzón-Quintana et al., 2021), acetic acid (Zheng et al., 2016; Huang et al., 2022), and other microbial fermentation processes. It is an advanced type of vinegar product (Chen et al., 2013, 2015, 2023; Yi et al., 2017). Guangxi is the largest sugarcane cultivation and production area in China, accounting for 60% of the total national area and 70% of the total national yield of sugarcane, which plays a major role in the growth and development of Chinese sugar agroindustries (Chen et al., 2020, 2023). The impact on the distribution of heavy metals in the soil has been continuously strengthened, and the content of heavy metals in the soil is increasing day-by-day. Since heavy metals are not degraded by microorganisms, they are not easy to move and accumulate continuously. It causes soil pollution and degrades crop yield and quality (Guga et al., 2023).

Based on previous studies, sugarcane original vinegar (SOV) preserves functional nutrients and nutritional rich values. The major component is acetic acid, and it also contains various organic acids such as oxalic, succinic, and tartaric acids (Chen et al., 2020), as well as flavonoids and phenols. Substances, such as benzoic, ferulic, quinic, chlorogenic, apigenin, kaempferol, caffeic, luteolin, p-coumaric, and other phenolic acids (He et al., 2017), and rich in different kinds of amino acids (Lin et al., 2022) with certain functionality. As an advanced type of vinegar, there are limited studies available on the safety and functional evaluation of sugarcane vinegar. This experiment mainly assesses and analyzes the efficacy and functional mechanisms of sugarcane vinegar to provide for the subsequent intensive processing and utilization of SOV and high-quality products.

## Materials and methods

### Experimental materials

A total of 220 specific pathogens, healthy Kunming mice, half male and half female (average weight: 18–35 g), were purchased from the Changsha Tianqin Biotechnology Co., Ltd., China [Experimental animal production license number: SCXK (Xiang) 2019-0014, quality certificate number: 430726201100369386, Hunan, China, and experimental animal center of Guangxi Medical University, laboratory animal production license number: SCXK (Gui) 2020-0004, quality certificate number: 0001734, Guangxi, China].

Sugarcane original vinegar (500 ml/bottle) is produced by the Agricultural Products Processing Research Institute of Guangxi Academy of Agricultural Sciences/Guangxi Key Laboratory of New Technology for Fruit and Vegetable Storage and Processing, according to DB 45/T 1536-2017 Guangxi Development and Preparation of Local Standards (Guangxi, China) (Bureau of Quality and Technical Supervision of Guangxi Zhuang Autonomous Region, 2017). Five histidine-deficient strains of *Salmonella typhimurium*, i.e., TA97a, TA98, TA100, TA102, and TA1535, were purchased from the MOLTOX Company, United States.

Mouse feed (basic material) was obtained from the Guangxi Experimental Animal Center, Guangxi Medical University, Nanning, Guangxi, China. High-fat feed (basic feed:lard:egg yolk powder:cholesterol:sodium cholate = 78.8:10:10:1:0.2) was self-made by the Institute of Agricultural Products Processing, Guangxi Academy of Agricultural Sciences/Guangxi Key Laboratory of New Technology for Fruit and Vegetable Storage and Processing.

Ethical approval (GXAAS/AEEIF/00001) was obtained, and animal experiments were performed according to the guidelines of the Animal Care and Use Committee (ACUC) of the Guangxi Academy of Agricultural Sciences, Nanning, Guangxi, China.

### Preparation of sugarcane original vinegar

Sugarcane is squeezed by press to obtain sugarcane juice (10–20°Brix). Filter equipment with 75–150 μm diameters (100–200 mesh) was used to filter the squeezed juice to meet the requirement of no impurities, and pasteurization was used to sterilize (sterilization temperature and time: 60–80°C and 20–30 min).

### Alcoholic and acetic acid fermentation

Sterilized cane juice was injected using the sterile fermentation equipment, activated fruit wine yeast (0.05–0.10% mass volume active dry yeast) was then added, stirred, and mixed thoroughly; the tank was sealed for fermentation process at an appropriate temperature (30–35°C); and fermentation continued until the volume percentage of alcohol content reached ≥4%.

Amended activated acetic acid bacteria (0.05%−0.10% active acetic acid bacteria in mass volume ratio) are added to the alcohol fermentation mash, stirred well, and mixed thoroughly, and then allowed to ferment to total acid (measured as acetic acid) of ≥3.5 g/100 ml and alcohol concentration of ≤ 0.1% by volume.

**Figure 1:**
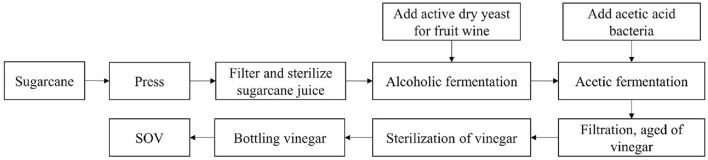
Flowchart of sugarcane original vinegar (SOV) fermentation process.

A filter device with a pore size of 0.22–0.45 μm was used and it was then transferred to the aging equipment at room temperature for up to 30 days. Ultra-high temperature instantaneous sterilization (sterilization temperature ≥121°C, sterilization time ≥ 6s) and aseptic filling equipment (Figure 1) were used.

### Physical and chemical indicators

The physico-chemical properties of SOV are shown in Table 1.

**Table 1:** The physical and chemical properties of sugarcane original vinegar.

**Physical and chemical index**	**Content**
Total acid (g/100 ml, citric acid)	3.65
Soluble solids (%)	11.5
Total sugar (g/L, glucose)	4.7
Lead (mg/L) detection limit (5 μg/L)	0.01

### Feeding conditions

The animal experiments were demonstrated in the Toxicology Laboratory of the Guangxi Zhuang Autonomous Region Center for Disease Control and Prevention, and the Experimental animals were raised in a barrier system with a temperature of 22–25°C and a relative humidity of 55%−70% [License number: SYXK (Gui) 2016-0002]. The irradiated animal feed is produced by Beijing Keao Xieli Feed Co., Ltd., China [License number: SCXK (Beijing) 2014-0010].

### Analysis of acute oral toxicity in mice

Using Horn's method (Standardization Administration of China, 2014a), 10 mice (18–22 g of body weight) in each group, including male and female (at half capacity of each), were administered different doses including 46,400, 21,500, 10,000, and 4,640 mg/kg BW. Prior to the experiment, the animals were fasted for 4 h with no restriction to drinking water. An amount of 50.0 and 23.2 g of SOV were weighed, respectively; pure water was added to them to prepare 100 ml; and the solutions were mixed well and made up to 500 and 232 mg/ml SOV solutions.

An amount of 46,400 mg/kg BW dose was applied to animals twice at a volume of 0.47 ml/20 g BW (time interval: 4 h), and the 10,000 and 4,640 mg/kg BW doses were 500 and 232 mg/ml, respectively, in a volume of 0.40 ml/20 g BW (vinegar @ 1 time). Observe the toxicity responses of the animals after treatment application. The animals were weighted once a week for up to 2 weeks. At the end of the experiment, the animals were selected for general observation. The acute toxicity of the test substance of SOV was evaluated according to the toxicity classification standard.

### Determination of bacterial reverse mutation (Ames)

Five histidine-deficient *S. typhimurium* strains, such as TA97a, TA98, TA100, TA102, and TA1535, were identified with the requirements for the analytical assessment. Five test groups, i.e., 5,000, 1,581, 500, 158, and 50 μg/dish, were set up, as well as a spontaneous reversion control group, a water solvent control group, a dimethyl sulfoxide (DMSO) solvent control group, and a positive mutagen control group. The conditions of the spontaneous back-change control group were the same as those of the sample group, except there was no sample. An amount of 50.0 and 15.81 g of SOV were weighed; each of them was gradually diluted with sterile water according to a specific ratio of 5.0, 0.5, 0.05, 1.581, and 0.158 mg/ml concentration; and then the solutions were sterilized by autoclaving (0.103 MPa, 20 min).

Rat liver microsomal enzyme (S9) induced by PCBs was used as an *in vitro* metabolic activation system. According to GB15193.4-2014 (Standardization Administration of China, 2014b), the plate incorporation method was adopted, and 0.1 ml of the test strain enrichment solution, 0.1 ml of the test substance solution, and 0.5 ml of the S9 mixture (when metabolic activation is required) were mixed and poured into the bottom medium plate. For the solvent control, sterilized pure water and DMSO were used to replace the samples, and the remaining conditions were the same as the sample group. Each strain of each group was prepared in three parallel dishes; they were incubated at 37°C for 48 h, and the number of colonies per plate was counted. If the number of reverted mutant colonies in each group of the test substance is upregulated in the dose–response relationship, or at least one strain has a reproducible increase in the number of reverted mutant colonies per plate at one or more doses under the different conditions, or no metabolic activation system is upregulated, that is, a positive mutagenesis test. The whole set of experiments was repeated three times under the same conditions.

### Analysis of mammalian erythrocyte micronucleus

A total of 50 Kunming mice were selected randomly (weighing 25–30 g) and divided into five groups, with 10 mice in each group (equally divided as male and female). According to the method of GB15193.5-2014 (Standardization Administration of China, 2014c), the experiment was carried out by oral treatment twice at a specific time interval (24 h). The test dosage, according to the LD_50_ results of the mice, was set at 10,000, 5,000, and 2,500 mg/kg BW for male mice and 7,350, 3,675, and 1,838 mg/kg BW for female mice. We used pure water as a negative control, and 40 mg/kg BW dose of cyclophosphamide (cp) was used as a positive control. An amount of 50.00, 25.00, and 12.50 g of samples were weighed, distilled water (100 ml) was added and mixed thoroughly, and samples of 500, 250, and 125 mg/ml were prepared for male mice. Then, an amount of 36.75, 18.38, and 9.19 g of samples were weighed, pure water (100 ml) was added and mixed thoroughly, and samples of 367.5, 183.8, and 91.9 mg/ml were prepared for female mice; then the animals were given intragastric administration at a volume of 0.4 ml/20 g BW, and an equal volume of distilled water was given to the negative control group. The positive control group was intraperitoneally injected with 4 mg/ml cyclophosphamide solution (prepared with normal saline) at a volume of 0.2 ml/20 g BW. After 24 h of the second sampling, the animals were killed by cervical dislocation, and the sternal bone marrow was diluted with calf serum for smears, dried naturally, fixed with methanol, stained with Gimsa application solution for 10 min, and washed with phosphate buffer solution (pH 6.8). After natural drying, the sample was observed under the optical microscope, polychromatic erythrocytes (PCE) per animal were counted as 2,000, and the micronucleus rate was expressed as the percentage of PCE containing micronuclei. At the same time, by counting and observing the positive staining of 200 PCE Red blood cells (NCE), the ratio of polychromatic erythrocytes/total erythrocytes (RBC) was calculated.

### Analysis of chromosomal aberrations in spermatogonial cells

According to GB 15193.8-2014 (National Standards Administration of China, 2014), 30 Kunming male mice with an average body mass of 25–35 g were selected and randomly divided into five groups, of which 10 were in one group and other four groups had five mice. The three doses of the test groups were 10,000, 5,000, and 2,500 mg/kg BW. Distilled water was applied as a negative control and cyclophosphamide (cp) of 40 mg/kg BW was used as a positive control. Distilled water (40 ml) was added, mixed thoroughly, and made up to different concentrations, i.e., 500, 250, and 125 mg/ml, and the animals were intragastrically applied with a volume of 0.4 ml/20 g BW as a negative control group. The positive control group was applied as a 2 mg/ml cyclophosphamide solution at a volume of 0.4 ml/20 g BW.

The animals in the high concentration were divided into two batches and killed 24 and 48 h after treatment, and the remaining groups were killed 24 h after treatment application. Four hours before treatment, each group was intraperitoneally injected with colchicine at a concentration of 0.5 mg/ml (equivalent dose of 5 mg/kg BW) at a volume of 0.2 ml/20 g BW. The animals were euthanized by cervical dislocation, the testes on both sides were taken, and the testes were placed in 1% trisodium citrate to separate the seminiferous tubules, and then fixed twice in fixative solution (methanol: glacial acetic acid = 3:1), at 60% glacial acetic acid (GAA) to soften and centrifuge. The collected cell suspension was dropped on chilled glass slides, made into 2–3 slices, dried in the air, stained by Giemsa, and examined by an optical microscope. A total of 100 metaphase cells were counted from each animal, the chromosome number and structural changes of spermatogonia were observed, and another 1,000 spermatogonia was observed to determine the mitotic index. The number of cells containing chromosomal structural aberrations and the number of chromosomal aberrations per cell in each animal were observed, and the number and frequency of different types of chromosomal structural aberrations in each group (chromosomal gaps are not included in the aberration rate) were counted.

### Determination of mammalian bone marrow cell chromosomal aberration

According to GB 15193.6-2014 (China National Standards Administration Committee, 2014), a one-time exposure method was adopted, and the samples were collected two times. A total of 100 Kunming mice (average body mass of 25–35 g, half male and half female) were selected. According to the LD_50_ results of the mice, the test doses of 10,000, 5,000, and 2,500 mg/kg BW for male mice and 7,350, 3,675, and 1,838 mg/kg BW for female mice were selected. We used distilled water as a negative control and 40 mg/kg BW of cyclophosphamide (cp) as a positive control. At each sampling time, there were five mice of each sex in each group (weighing 50, 25, and 12.50 g). Distilled water (100 ml) was added and mixed thoroughly, and 500, 250, and 125 mg/ml solutions for male mice were prepared, weighing 36.75, 18.38, and 9.19 g. Distilled water (100 ml) was added and mixed thoroughly, and 367.5, 183.8, and 91.9 mg/ml solutions for female mice were prepared. Then, the animals were intragastrically applied with a volume of 0.4 ml/20 g BW. The negative control group was applied as the same volume of distilled water, and the positive control was infused with an equal volume of 2 mg/ml cyclophosphamide solution.

Animals in each group were divided into two subgroups. Subgroup 1 was euthanized 18 h after exposure to collect the first specimen, and subgroup 2 was euthanized 24 h after the animals in subgroup 1 to collect the second sample for analysis. Four hours prior to treatment, each group was intraperitoneally injected with colchicine at a concentration of 0.4 mg/ml (equivalent dose of 4 mg/kg BW) at a volume of 0.2 ml/20 g BW. Animals were euthanized by cervical dislocation; the femur was taken out, and the epiphyses at both ends were cut off. The bone marrow was washed into a centrifuge tube with 5 ml of normal saline and blown to make a cell suspension, centrifuged at 1,000 r/min for 10 min, and the supernatant was discarded. A volume of 0.075 mol/L potassium chloride was added (7 ml) and the cells were mixed gently with a dropper and then placed in a 37°C water bath for hypotonic treatment for 15 min. Next, 2 ml of fixative solution (methanol:glacial acetic acid = 3:1) was added, mixed thoroughly, and centrifuged at 1,000 r/min (10 min), and the supernatant was discarded. A volume of 7 ml of fixative was added, fixed for 15 min, and centrifuged at 1,000 r/min (10 min); the supernatant was discarded; and the whole process was repeated once. The cell suspension was dropped on ice water slides, made into 2–3 slices, dried in the air, stained by Giemsa, and examined by an optical microscope. One hundred metaphase cells were counted for each animal, observed the chromosome number and structural changes of bone marrow cells, and observed another 1,000 bone marrow cells to determine the mitotic index. The number of cells with chromosomal structural aberrations in each animal was recorded. The number of different types of chromosomal structural aberrations in each group was counted (chromosomal gaps were not included in the aberration rate), and the chi-square test was performed on the rate of chromosomal structural aberrations.

### Functional verification

A total of 60 28-day-old SPF male mice with similar body mass were selected, fed basal feed for 7 days, then randomly divided as normal saline (control group, MG group, i.e., fed with high-fat feed and gavaged with normal saline) and SOV dosage group (TG, i.e., fed with high-fat feed and gavaged with raw vinegar), weighed once a week, and observed for general characteristics such as mental activity, hair changes, water intake, food intake, and excrement volume. The mice were fed continuously for 8 weeks, and the body mass and serum profile were measured (Li et al., 2020b,c).

### Data analysis

The SPSS 13.0 software was used for one-way analysis of variance and pairwise comparison, and the data were expressed as mean ± standard deviation (SD). The significant differences at *P* < 0.05 and *P* < 0.01 levels were analyzed. Graphics were prepared using the Origin 2021 (Origin Lab, Northampton, MA) software.

## Results and discussion

### Acute oral toxicity

The results of the acute toxicity test for each group are shown in Table 2. After 0.5 h of treatment, it was found that the test animals in the 4,640 mg/kg BW group appeared sluggish, unresponsive, and died, and the death time was after giving the test substance (2–96 h). Autopsies of dead animals showed varying degrees of gastric effusion and flatulence. It may be related to the side effects of vinegar. After vinegar is used, it will change the pH of the local environment in the body. Vinegar can cause the digestive organs to secrete a large amount of digestive juice, and vinegar will corrode the gastrointestinal mucosa and aggravate the development of ulcer disease (Budak et al., 2014; Luo and Xu, 2019). The higher concentration of raw sugarcane vinegar stimulates the digestive organs. At the end of the experimentation, the surviving animals were dissected, and there was no abnormality in the general observation. This is consistent with the research results of our research group's previous research on the effects of SOV on the internal organs of mice fed high-fat diets. There was no difference in the color, size, or character of the organs, and feeding SOV had no adverse effects on the organs of mice (Li et al., 2020a). The median lethal dose (LD_50_) was carried out due to animal death, and the acute oral toxicity LD_50_ of SOV in male and female mice was 20,000 and 14,700 mg/kg BW, respectively. The LD_50_ of female mice is lower than male mice, and female mice are more sensitive to the test substance than male mice, which is consistent with the acute toxicity study of *Ganoderma leucocontextum* (Deng et al., 2023). According to the acute toxicity dose grading standard and research results in GB15193.3-2014, the acute oral toxicity of raw SOV belongs to the actual non-toxic level, and it is not easy for people with hyperacidity and gastric ulcers to use high concentrations (Younes et al., 2022).

**Table 2:** Acute toxicity analytical results of SOV on mice.

**Category**	**Dose group (mg/kg BW)**	**Number of animals**	**Body weight (g)**				
			**0 days**	**7 days**	**Body weight gain (%)**	**14 days**	**Body weight gain (%)**
Male	46,400	5	20.4 ± 1.4	–	–	–	–
	21,500	5	20.4 ± 1.3	25.8 ± 1.1	26.47	29.8 ± 1.2	46.08
	10,000	5	20.1 ± 1.2	25.4 ± 1.1	26.37	29.5 ± 1.2	46.77
	4,640	5	20.4 ± 1.3	26.1 ± 1.5	27.94	30.8 ± 1.8	50.98
Female	46,400	5	20.1 ± 1.5	–	–	–	–
	21,500	5	19.8 ± 1.3	24.5 ± 1.6	23.74	28.2 ± 1.1	42.42
	10,000	5	19.7 ± 1.4	24.3 ± 1.1	23.35	28.3 ± 1.3	43.65
	4,640	5	19.9 ± 1.3	25.3 ± 1.4	27.14	29.5 ± 1.5	48.24

All data are indicated as mean ± SD. LD50: 20,000 mg/kg BW for male mice, 95% credible limit: 12,300–32,400 mg/kg; BW: 14,700 mg/kg BW for female mice, 95% credible limit: 8,620–25,000 mg/kg BW.

### Responses to bacterial reverse mutation

Positive controls such as TA97a+S_−9_, TA98+S_−9_, and TA100+S_−9_ used 2-aminofluorene (10 μg/dish); TA98–S_−9_ used daunomycin (6 μg/dish); TA97a–S_−9_ and TA102–S_−9_ adopted dexazone (50 μg/dish); TA100–S_−9_ and TA1535–S_−9_ adopted sodium azide (1.5 μg/dish); TA102+S_−9_ adopted 1, 8-dihydroxyanthraquinone (50 μg/dish); and TA1535+S_−9_ adopted cyclophosphamide (200 μg/dish; Tables 3, 4).

**Table 3:** Result findings of the first bacterial reverse mutation test of SOV (mean ± SD).

**Dose group (μg/plate)**	**TA97a**		**TA98**		**TA100**		**TA102**		**TA1535**	
	**−S_−9_**	**+S_−9_**	**−S_−9_**	**+S_−9_**	**−S_−9_**	**+S_−9_**	**−S_−9_**	**+S_−9_**	**−S_−9_**	**+S_−9_**
5,000	136.0 ± 28.0	126.3 ± 28.5	39.7 ± 4.2	40.3 ± 0.6	146.7 ± 18.6	160.0 ± 33.2	266.0 ± 29.6	296.0 ± 22.5	25.3 ± 7.1	28.0 ± 6.6
1,581	107.7 ± 11.7	129.7 ± 30.1	35.7 ± 6.4	38.3 ± 3.8	143.0 ± 13.9	169.3 ± 25.5	285.0 ± 11.0	274.0 ± 23.3	18.0 ± 2.7	19.0 ± 1.0
500	132.0 ± 17.4	150.7 ± 47.3	44.3 ± 4.9	45.7 ± 3.8	176.3 ± 24.4	166.7 ± 16.4	293.3 ± 31.9	267.7 ± 31.0	26.3 ± 6.0	21.3 ± 3.5
158	129.0 ± 39.0	139.0 ± 21.4	38.3 ± 5.5	40.7 ± 4.2	172.3 ± 24.5	157.3 ± 31.6	267.0 ± 33.1	278.7 ± 29.0	23.3 ± 5.1	29.7 ± 1.5
50	129.0 ± 38.7	143.3 ± 32.0	40.3 ± 4.9	40.7 ± 5.5	186.0 ± 15.7	178.0 ± 3.0	267.0 ± 16.6	265.3 ± 20.4	23.7 ± 4.2	20.3 ± 6.7
Spontaneous change back	134.3 ± 37.1	135.7 ± 22.5	34.0 ± 2.0	37.0 ± 4.0	149.7 ± 25.6	169.7 ± 42.3	288.0 ± 23.6	300.3 ± 16.9	30.0 ± 3.0	28.0 ± 5.3
Water control	124.3 ± 42.4	140.7 ± 30.6	40.0 ± 1.0	41.7 ± 3.8	154.7 ± 36.9	143.7 ± 13.6	293.7 ± 21.1	289.3 ± 21.9	24.7 ± 3.2	28.7 ± 4.9
DMSO	117.0 ± 21.8	144.7 ± 44.1	35.3 ± 6.1	39.3 ± 4.5	173.3 ± 29.3	169.3 ± 15.5	274.3 ± 35.6	283.0 ± 29.5	32.7 ± 1.2	30.7 ± 3.5
Positive control	2,544.0 ± 94.3	1,724.0 ± 780.1	2,870.7 ± 146.6	4,300.0 ± 191.2	2,940.0 ± 151.2	2,745.3 ± 158.7	757.7 ± 36.6	803.0 ± 28.1	319.7 ± 340.7	141.7 ± 190.1

The above results (number of colonies) are the mean ± standard deviation (*n* = 3).

Positive control: TA97a + S-9, TA98 + S-9, and TA100 + S-9 use 2-aminofluorene (10 μg/dish); TA98–S-9 use daunomycin (6 μg/dish); TA97a–S-9 and TA102–S-9 were treated with dexazone (50 μg/dish). TA100-S-9 and TA1535-S-9 were treated with sodiumazide (1.5 μg/dish). TA102 + S-9 were treated with 1,8-dihydroxyanthraquinone (50 μg/dish) and TA1535 + S-9 adopts cyclophosphamide (200 μg/dish).

**Table 4:** The analytical observations of the second bacterial reverse mutation test of SOV (mean ± SD).

**Dose group (μg/dish)**	**TA97a**		**TA98**		**TA100**		**TA102**		**TA1535**	
	**−S_−9_**	**+S_−9_**	**−S_−9_**	**+S_−9_**	**−S_−9_**	**+S_−9_**	**−S_−9_**	**+S_−9_**	**−S_−9_**	**+S_−9_**
5,000	115.7 ± 10.8	130.0 ± 15.6	40.0 ± 4.4	39.0 ± 6.0	171.7 ± 36.9	184.3 ± 10.1	272.0 ± 25.5	270.7 ± 15.5	27.0 ± 3.0	24.3 ± 4.9
1,581	122.0 ± 27.8	151.0 ± 15.1	42.0 ± 3.0	38.3 ± 4.2	147.0 ± 15.5	177.0 ± 18.1	283.0 ± 6.1	287.3 ± 23.2	28.3 ± 6.4	26.0 ± 3.5
500	137.3 ± 27.3	126.0 ± 13.8	40.3 ± 3.5	43.0 ± 2.7	161.0 ± 9.0	155.7 ± 37.1	267.7 ± 25.2	278.7 ± 18.2	23.7 ± 2.5	24.7 ± 4.7
158	130.7 ± 23.6	131.3 ± 25.5	40.7 ± 3.5	42.0 ± 2.7	155.3 ± 27.5	147.3 ± 23.1	282.7 ± 32.5	263.7 ± 20.8	23.7 ± 6.7	26.7 ± 4.0
50	129.7 ± 20.4	160.3 ± 12.5	39.3 ± 4.5	43.7 ± 5.9	166.7 ± 9.1	137.3 ± 0.6	281.7 ± 10.5	252.7 ± 11.5	23.0 ± 3.0	24.0 ± 5.2
Spontaneous change	127.7 ± 13.8	112.0 ± 1.0	35.7 ± 3.1	41.7 ± 5.1	168.0 ± 14.4	132.7 ± 8.7	262.0 ± 29.7	287.7 ± 28.4	21.0 ± 3.0	22.0 ± 5.3
Water control	139.0 ± 28.6	135.7 ± 30.6	42.0 ± 3.6	43.7 ± 4.2	144.0 ± 10.4	138.3 ± 15.0	261.0 ± 18.7	281.7 ± 13.6	31.0 ± 2.0	27.7 ± 6.1
DMSO	126.7 ± 21.7	151.3 ± 21.1	40.0 ± 4.0	36.7 ± 3.8	186.0 ± 16.8	177.7 ± 5.0	288.0 ± 32.8	275.0 ± 17.6	22.7 ± 6.8	29.0 ± 4.6
Positive control	2,570.7 ± 153.7	1,781.3 ± 80.0	2,988.0 ± 137.6	4,104.0 ± 198.9	2,948.0 ± 115.3	2,852.0 ± 108.9	725.7 ± 33.3	819.0 ± 31.2	326.7 ± 29.1	155.3 ± 18.0

The above results (number of colonies) are the mean ± standard deviation of assessed by three plates.

Tables 3, 4 show the number of back-mutant colonies in the SOV bacterial back-mutation test. In the five test strains, such as TA97a, TA98, TA100, TA102, and TA1535, the number of reverted colonies in each concentration and group of sugarcane vinegar did not exceed the number of spontaneous reverted colonies after addition or not of the S9 metabolic activation system. The number of colonies was doubled, and there was no dose–response relationship between the number of back-changing colonies in each dose, indicating that SOV would not induce gene mutations in the test strains, while the positive control group showed obvious mutagenicity and reverse. The number of changed colonies was far more than 2-fold that of the spontaneous back-mutation group. The GB15193.4-2014 standard justified the mutagenesis negative tests, which means that it has no direct effect on the histidine-deficient strains of *S. typhimurium* (TA97a, TA98, TA100, TA102, and TA1535) or indirect mutagenesis.

### Impact of mammalian erythrocyte micronucleus

The statistical results of the incidence of polychromatic erythrocyte micronuclei in the bone marrow of the tested animals are shown in Table 5. At different doses of SOV, such as 10,000, 5,000, and 2,500 mg/kg BW, the micronucleus of polychromatic erythrocyte bone marrow in female and male mice was compared with the negative control group. There was no significant difference in the nuclear rate (*P* > 0.05), and the PCE/RBC values of each dose group were not < 20% of the negative control group, and there was no significant difference compared with the negative control group. The difference between the micronucleus rate of the control group (cyclophosphamide) and the negative control group was very significant (*P* < 0.01). According to the procedure of GB 15193.5-2014, within the range of doses, the results of this experiment showed that sugarcane vinegar would not enhance the rate of micronuclei in mammalian polychromatic erythrocytes. No damage or inhibition was detected.

**Table 5:** Effect of SOV on the incidence of micronucleus in mouse bone marrow polychromatic erythrocytes (mean ± SD).

**Gender**	**Dose group (mg/kg BW)**	**Number of animals**	**Number of tested PCEs (units)**	**Number of PCEs with micronuclei (units)**	**Micronucleus rate (%)**	**Number of tested PCEs (pcs)**	**Number of NCEs (number)**	**PCE/RBC (%)**
Male	10,000	5	5 × 2,000	16	1.6 ± 0.4	5 × 200	891	0.529 ± 0.015
	5,000	5	5 × 2,000	17	1.7 ± 0.3	5 × 200	888	0.530 ± 0.009
	2,500	5	5 × 2,000	15	1.5 ± 0.4	5 × 200	905	0.525 ± 0.017
	Negative control	5	5 × 2,000	15	1.5 ± 0.4	5 × 200	896	0.528 ± 0.017
	Positive 40 (cp)	5	5 × 2,000	216	21.6 ± 2.7^**^	5 × 200	1,008	0.498 ± 0.011
Female	7,350	5	5 × 2,000	14	1.4 ± 0.2	5 × 200	896	0.528 ± 0.012
	3,675	5	5 × 2,000	14	1.4 ± 0.4	5 × 200	893	0.528 ± 0.014
	1,838	5	5 × 2,000	17	1.7 ± 0.3	5 × 200	877	0.533 ± 0.008
	Negative control	5	5 × 2,000	14	1.4 ± 0.4	5 × 200	905	0.525 ± 0.005
	Positive 40 (cp)	5	5 × 2,000	208	20.8 ± 2.0^**^	5 × 200	989	0.503 ± 0.005

PCE/RBC is the ratio of polychromatic erythrocytes to total erythrocytes.

^**^Indicates a very significant difference at *P* < 0.01 compared with the negative control group.

cp, cyclophosphamide.

### Chromosomal aberration test in mouse spermatogonia

As mentioned in Table 6, compared with the negative control group, there was no significant difference in the number of chromosome aberrations, changes in chromosome structure, or rate of aberrant cells in the spermatogonia of mice in three different dose groups of SOV (10,000, 5,000, and 2,500 mg/kg BW; *P* > 0.05). There was no dose–response relationship or statistical significance among the dose groups. In addition, the mitotic index of the three different dose groups of SOV was not lower than 50% of the negative control group compared with the negative control group. There was no significant difference, but the difference in the chromosomal aberration cell rate between the cyclophosphamide positive control group and the negative control group was more significant (*P* < 0.01). According to the method of GB 15193.8-2014, within the concentration level, the results of this experiment showed that SOV does not have an aberrant effect on spermatogonia chromosomes in mice, and the results are negative.

**Table 6:** Effects of SOV on chromosomal aberrations in mouse spermatogonia (mean ± SD).

**Dose group (mg/kg BW)**	**Observe the number of cells (pcs)**	**Chromosome number change**		**Chromosomal structural changes** ^*^								**Distorted cell number (units)**	**Aberrant cell rate (%)**	**Mitotic index**
		**Aneuploidy**	**Polyploid**	**Fissure**	**Fracture**	**Piece**	**Minute body**	**Ring**	**Multiple centromere**	**Single swap**	**Unspecified**			
10,000 (24 h)	100 × 5	0	21	7	3	0	0	0	0	0	0	24	4.8 ± 0.4	6.9 ± 1.2
10,000 (48 h)	100 × 5	0	20	8	2	0	0	0	0	0	0	22	4.4 ± 1.3	7.5 ± 1.1
5,000	100 × 5	0	16	9	2	0	0	0	0	0	0	18	3.6 ± 0.5	7.6 ± 1.1
2,500	100 × 5	0	20	8	4	0	0	0	0	0	0	24	4.8 ± 0.8	7.8 ± 1.2
Negative control	100 × 5	0	21	8	3	0	0	0	0	0	0	24	4.8 ± 0.8	7.5 ± 1.4
Positive control	100 × 5	0	23	24	20	19	2	1	0	0	0	65	13.0 ± 2.5^**^	4.4 ± 0.6

^*^Circular changes of structural changes include centromere and non-centromere. Multiple centromere refers to more than two centromeres, cracks are not included in the number of aberrant cells, and the rate of aberrant cells = number of aberrant cells/number of observed cells × 100.

^**^Indicates that compared with the negative control group, *P* < 0.01.

### Mammalian bone marrow cell chromosome aberration test

The bone marrow cell chromosomal aberration test is different from the bone marrow micronucleus test. Cell chromosomal aberration in interphase and prophase, thus directly reflecting the incidence of cell chromosomal aberration and judging whether the test substance has a mutation effect (de Barros et al., 2022; Deng et al., 2023). As mentioned in Tables 7, 8, the first and second sampling time results, SOV in three different dose levels, the number of chromosome aberrations, chromosome structure changes, and aberrant cell rates in bone marrow cells of female and male mice were compared with the negative control group. There was no significant difference (*P* > 0.05), no dose–response relationship between the dose groups, and no statistical significance. In addition, three different dose groups of SOV were administered to female and male mice. The mitotic index of bone marrow cells was not lower than 50% of the negative control group, and there was no significant difference compared with the negative control group. While the difference in the rate of chromosomal structural aberration cells between the cyclophosphamide positive and the negative control groups was significant (*P* < 0.01). According to the method of GB 15193.6-2014, within the dosage range, the results of this experiment showed that the SOV would not have an aberrant effect on the chromosomes of bone marrow cells in mice.

**Table 7:** The effect of the first sampling of raw SOV on chromosomal aberrations in mouse bone marrow cells (mean ± SD).

**Gender**	**Dose group (mg/kg BW)**	**Observe the number of cells (pcs)**	**Chromosome number change**			**Chromosomal structural changes** ^*^										**Mitotic index**
			**Aneuploidy**	**Polyploid**	**Endoduplication**	**Fracture**	**Minute body**	**Doublemicrosome**	**Filamentous point ring**	**Acentric ring**	**Single swap**	**Fissure**	**Non-characteristic type**	**Aberrant cells (units)**	**Structural distortion cell rate (%)**	
Male	10,000	100 × 5	0	1	0	7	1	0	0	0	0	1	1	9	1.8 ± 0.8	7.6 ± 1.1
	5,000	100 × 5	0	2	0	5	0	0	0	0	0	2	1	6	1.2 ± 0.8	7.3 ± 1.2
	2,500	100 × 5	0	1	0	4	0	0	0	0	1	1	2	7	1.4 ± 1.5	7.9 ± 1.2
	Negative control	100 × 5	0	3	1	6	0	0	1	0	1	3	1	9	1.8 ± 0.8	7.8 ± 1.7
	40 (cp)	100 × 5	0	6	1	72	1	0	1	0	0	38	2	76	15.2 ± 3.3^**^	4.8 ± 1.1
Female	7,350	100 × 5	0	3	0	5	0	0	1	1	0	1	1	8	1.6 ± 1.1	7.5 ± 1.2
	3,675	100 × 5	0	1	0	5	0	0	0	1	0	1	2	8	1.6 ± 1.5	7.6 ± 1.0
	1,838	100 × 5	0	3	0	3	0	0	1	0	0	3	2	6	1.2 ± 0.8	7.8 ± 1.2
	Negative control	100 × 5	0	1	0	3	0	0	1	1	0	3	1	6	1.2 ± 0.8	8.0 ± 1.3
	40 (cp)	100 × 5	0	8	0	76	0	0	1	1	1	47	3	82	16.4 ± 1.5^**^	5.0 ± 1.4

^*^Cracks are not included in the number of structurally aberrant cells, the rate of structurally aberrant cells = the number of structurally aberrant cells/the number of observed cells × 100.

^**^Indicates that compared with the negative control group, *P* < 0.01.

**Table 8:** The effect of the second sampling of raw SOV on the chromosomal aberration of mouse bone marrow cells (mean ± SD).

**Gender**	**Dose group (mg/kg BW)**	**Observe the number of cells (pcs)**	**Chromosome number change**			**Chromosomal structural changes** ^*^										**Mitotic index**
			**Aneuploidy**	**Polyploid**	**Endoduplication**	**Fracture**	**Minute body**	**Doublemicrosome**	**Filamentous point ring**	**Acentric ring**	**Single swap**	**Fissure**	**Non-characteristic type**	**Aberrant cells (units)**	**Structural distortion cell rate (%)**	
Male	10,000	100 × 5	0	1	0	4	0	0	0	1	0	2	0	5	1.0 ± 0.7	7.5 ± 1.6
	5,000	100 × 5	0	2	0	4	1	0	0	0	0	2	4	9	1.8 ± 1.3	8.0 ± 1.4
	2,500	100 × 5	0	1	0	5	0	0	0	1	0	3	1	7	1.4 ± 1.7	7.9 ± 1.2
	Negative control	100 × 5	0	1	0	3	0	0	0	1	0	1	0	4	0.8 ± 0.8	7.3 ± 1.6
	40 (cp)	100 × 5	0	7	0	70	1	0	0	1	0	38	3	75	15.0 ± 4.1^**^	4.6 ± 1.3
Female	7,350	100 × 5	0	2	0	4	1	0	0	0	0	0	1	6	1.2 ± 0.8	8.2 ± 1.0
	3,675	100 × 5	0	3	0	4	1	0	0	1	0	1	1	7	1.4 ± 1.3	7.7 ± 1.9
	1,838	100 × 5	0	1	0	5	1	0	0	0	0	2	2	8	1.6 ± 1.5	7.9 ± 1.0
	Negative control	100 × 5	0	1	0	3	1	0	1	0	0	1	1	6	1.2 ± 0.8	7.8 ± 1.1
	40 (cp)	100 × 5	0	6	0	72	1	0	2	1	0	39	3	79	15.8 ± 3.2^**^	4.9 ± 1.2

^*^Cracks are not included in the number of structurally aberrant cells, and the rate of structurally aberrant cells = the number of structurally aberrant cells/the number of observed cells × 100.

^**^Indicates that compared with the negative control group, *P* < 0.01.

### Functional test verification

As shown in Table 9, after continuous observation for 8 weeks, the mental status of the mice in each group was normal for their activities. The mice had smooth fur; a good appetite; a plump body; agile activities; bright eyes; normal urine; no abnormal secretions from the nose, eyes, or mouth; and no adverse conditions. Mice fed a high-fat diet for 4 weeks were fed SOV continuously, and the body weight of the mice did not increase negatively, indicating that SOV has a weight-loss effect (Younes et al., 2022).

**Table 9:** Effects of SOV on the behavioral state and body weight of mice.

**Group**	**Extremities**	**Hair**	**Diet**	**Drinking water**	**Stool**	**Urine**	**Initial weight after modeling**	**Final weight**	**Weight gain**
Normal group (CG)	—	—	—	—	—	—	33.40 ± 0.84	37.40 ± 0.84	4.0 ± 0.74
Model group (MG)	—	—	—	—	—	—	36.60 ± 2.22	38.20 ± 3.33	1.90 ± 3.12
Original vinegar group (TG)	—	—	—	—	—	—	35.30 ± 2.45	37.10 ± 4.33	−6.60 ± 1.96^**^

“—” means normal.

^**^P < 0.01 compared with HF group.

**Figure 2:**
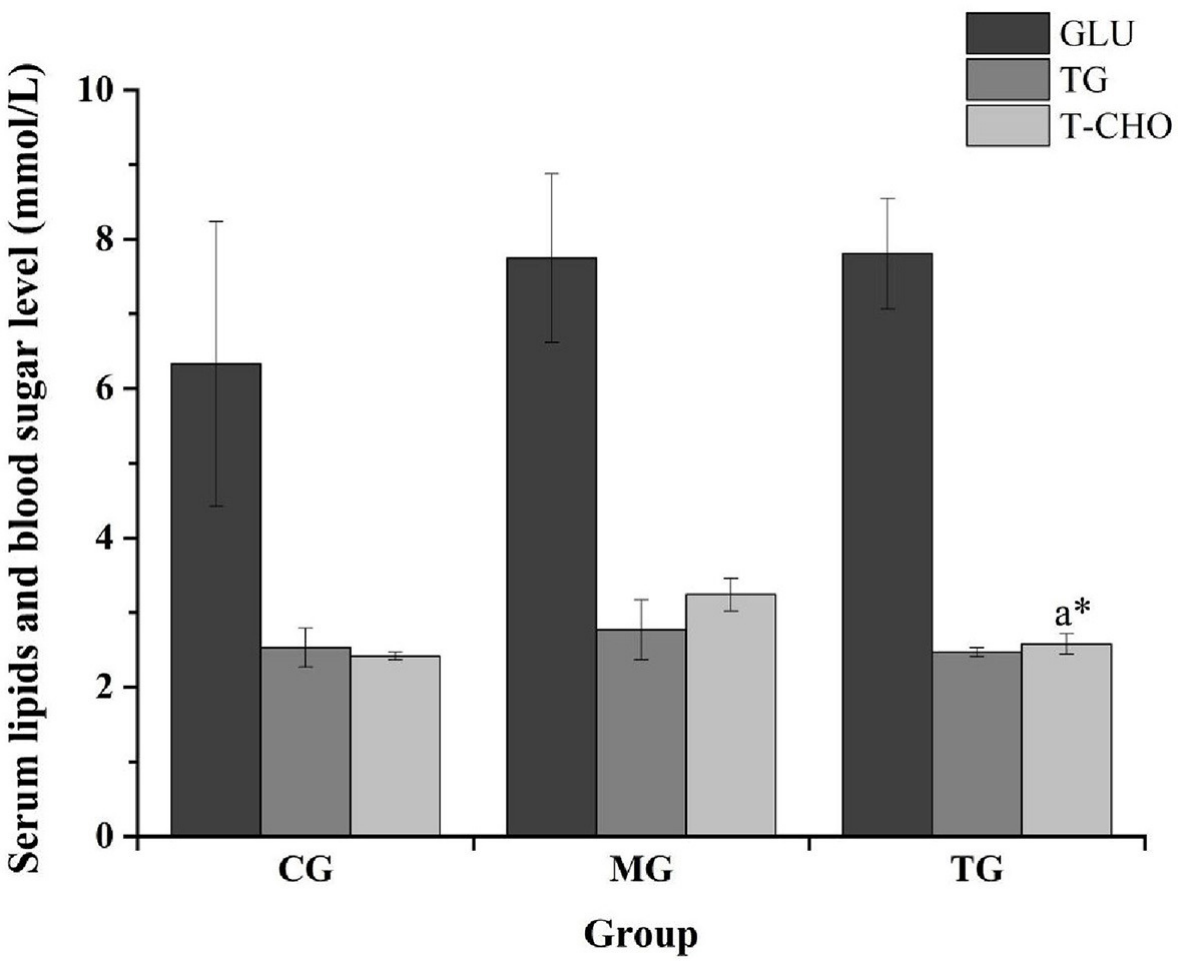
Responses of blood glucose and blood lipid levels in different groups. The ^*^ symbol indicates significance at *P* < 0.05.

**Figure 3:**
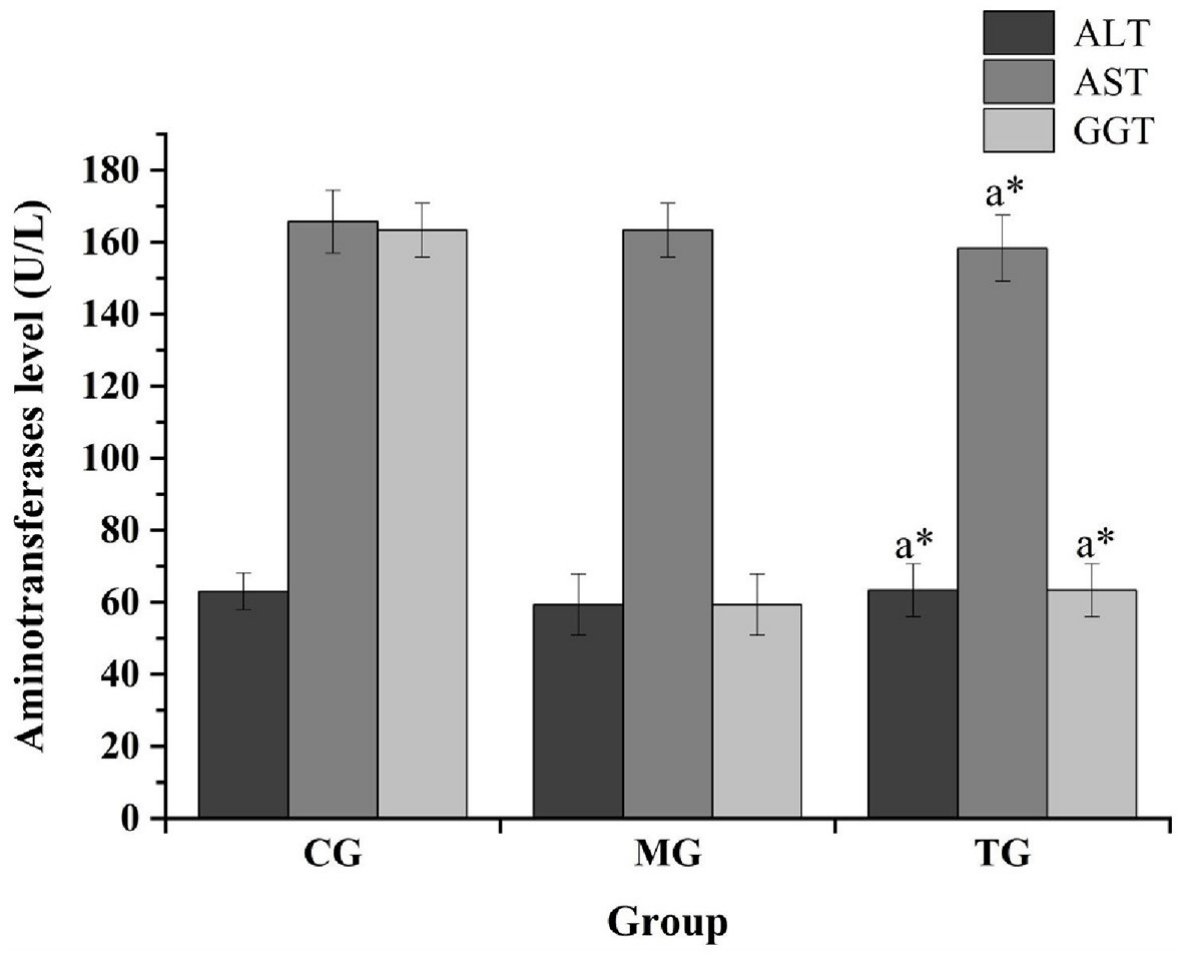
Impact of transaminase levels in different groups. The ^*^ symbol indicates significance at *P* < 0.05.

The routine blood analysis of the mice was performed, and the results are shown in Figures 2, 3 and Table 10. Compared with the normal group, the blood indicators of the mice fed the high-fat diet for 4 weeks were different in nine indicators. The degree of increase and the six indicators decline are different degrees. After the high-fat mice were fed with SOV for 4 weeks, compared with the model group MG, the levels of ALT and GCT in the serum of the mice were significantly increased (*P* < 0.05), compared with the normal group CG and the model group MG. The levels of GLU, TG, and T-CHO in mice in the TG group were slightly increased or decreased without significant changes. The levels of CREA, BUN, and UA in serum had no significant changes, which indicated that SOV did not cause kidney damage.

**Table 10:** Effects of SOV on serum biochemical indicators in mice.

**Group**	**Normal group (CG)**	**Model group (MG)**	**Original vinegar group (TG)**	**Raw vinegar group changes (TG vs. CG)**
ALT/(UL^−1^)	63.00 ± 5.42	59.33 ± 8.39	63.33 ± 7.61^*^	↑
AST/(U L^−1^)	165.67 ± 8.54	163.33 ± 7.52	158.33 ± 9.31^*^	↓
GGT/(U L^−1^)	163.33 ± 7.31	59.33 ± 8.64	63.33 ± 7.41^*^	↓
AST/ALT	3.07 ± 0.37	4.75 ± 0.49	3.31 ± 0.46	↑
TP/(g L^−1^)	67.50 ± 5.48	74.20 ± 3.42	71.60 ± 5.54	↑
ALB/(g L^−1^)	29.90 ± 2.49	29.60 ± 1.91	31.80 ± 0.16	↑
GLB/(g L^−1^)	37.53 ± 8.14	44.57 ± 4.41	39.83 ± 5.61	↑
ALB/GLB	0.81 ± 0.21	0.67 ± 0.18	0.79 ± 0.10	↓
TBIL/(μmol L^−1^)	0.30 ± 0.48	0.70 ± 0.49	1.67 ± 0.60	↑
CREA/(μmol L^−1^)	21.00 ± 0.48	23.00 ± 1.59	22.00 ± 4.51	↑
UA/(μmol L^−1^)	105.30 ± 9.79	94.00 ± 4.93	104.20 ± 8.39^*^	↓
BUN/(mmol L^−1^)	8.33 ± 0.58	6.67 ± 1.16	7.67 ± 0.56^*^	↓
GLU/(mmol L^−1^)	6.33 ± 1.91	7.75 ± 1.13	7.81 ± 0.76	↑
TG/(mmol L^−1^)	2.53 ± 0.26	2.77 ± 0.40	2.47 ± 0.06	↓
T-CHO/(mmol L^−1^)	2.42 ± 0.05	3.24 ± 0.22	2.58 ± 0.13^*^	↑

The ↓, ↑, and ^*^ symbols indicate decrease and increase values, and ^*^ is statistically significant at *P* < 0.05.

The previous study of our research group found that SOV fermented with sugarcane juice has strong antioxidant activities (Zheng et al., 2015), and SOV can significantly reduce the body mass of mice fed a high-fat diet and can improve blood lipids, reduce blood sugar concentration, and promote fat loss. The effect of reducing blood lipid levels can reduce the levels of triglycerides, total cholesterol, and low-density lipoprotein cholesterol in the plasma of high-fat mice. At the same time, the ability to resist oxidative stress can reduce amylase activity and increase lipase activity, increase the activity of superoxide dismutase (SOD) and glutathione peroxidase (GSH-Px) in plasma and liver, and control the further development of obesity and its complications (Li et al., 2020a,b,c).

## Conclusion

Sugarcane vinegar is a healthy and wealthy product with different characteristics, such as antidiabetic, antihyperlipidemic, antimicrobial, and anti-inflammatory effects. Different approaches have unraveled the presence of different bioactive compounds in sugarcane vinegar, which are associated with the raw material used to produce vinegar. The test results showed that SOV products contained some heavy metals; the acute oral toxicity LD_50_ of SOV in male and female mice was 20,000 and 14,700 mg/kg BW, respectively. Oral toxicity is practically non-toxic. The results of genetic toxicity tests (bacterial reverse mutation, mammalian erythrocyte micronucleus, mouse spermatogonial chromosomal aberration, and mouse bone marrow cell chromosomal aberration test) showed that SOV had no damage or inhibitory effect on bone marrow erythrocytes in mice. It has no mutagenic or aberrant effect on the chromosomes of bone marrow cells or spermatogonia in mice. Raw SOV is rich in nutrition, organic acids, flavonoids, phenolic acids, and amino acids. Adhikari et al. (2013) found that SOV has a lipid-lowering effect after drinking it for patients with hyperlipidemia in clinical practice. It has the effect of increasing HDL (Adhikari et al., 2013). For the development and high-value utilization of SOV in the next step, such as the development of high-end products such as SOV beverages, functional SOV powders, and SOV capsules, provide technical direction and safety, and improve the high-quality and sustainable development of the sugarcane industry.

## Data availability statement

The original contributions presented in the study are included in the article/supplementary material, further inquiries can be directed to the corresponding author.

## Ethics statement

The animal study was reviewed and approved by Ethical approval (GXAAS/AEEIF/00001) was obtained and animal experiments were performed in accordance with the guidelines of the Animal Care and Use Committee (ACUC) of Guangxi Academy of Agricultural Sciences, Nanning, Guangxi, China.

## Author contributions

F-JZ, BL, and G-LC conceived and designed the experiment, supervised, and drafted the original manuscript. BL, Y-XY, X-CF, and KKV performed experimental analysis. All authors have read and approved the final manuscript for publication.
