# Corrigendum: Efficacy and functionality of sugarcane original vinegar on mice

**DOI:** 10.3389/fmicb.2023.1281182

**Published:** 2023-09-05

**Authors:** Feng-Jin Zheng, Bo Lin, Yu-Xia Yang, Xiao-Chun Fang, Krishan K. Verma, Gan-Lin Chen

**Affiliations:** ^1^Institute of Agro-Products Processing Science and Technology, Guangxi Academy of Agricultural Sciences, Nanning, Guangxi, China; ^2^Guangxi Key Laboratory of Fruits and Vegetables Storage-Processing Technology, Nanning, China; ^3^Key Laboratory of Sugarcane Biotechnology and Genetic Improvement (Guangxi), Ministry of Agriculture and Rural Affairs, Guangxi Key Laboratory of Sugarcane Genetic Improvement, Sugarcane Research Institute, Guangxi Academy of Agricultural Sciences, Nanning, Guangxi, China; ^4^School of Chemistry and Chemical Engineering, Guangxi Minzu University, Nanning, Guangxi, China

**Keywords:** sugarcane original vinegar, safety, functionality, evaluation, mice

In the published article, there was a mistake in the keyword “raw sugarcane vinegar”.

The correct keyword is “sugarcane original vinegar”.

In the published article “Chen et al. 2015b” was cited in the Introduction, first-paragraph, line 5 as “It is an advanced type of vinegar product (Chen et al., 2015a,b, 2023; Yi et al., 2017)”.

The correct citation is “It is an advanced type of vinegar product (Chen et al., 2013, 2015, 2023; Yi et al., 2017)”.

In the published article, the reference “Chen, G.-L., Zheng, F.-J., Lin, B., Yang, Y.-X., Fang, X.-C., Verma, K. K., et al. (2015b). Research on the production technology of sugarcane vinegar and fruit vinegar beverages. *China Brew*. 34, 154–157” was incorrectly cited.

**The correct reference is:** “Chen, G.-L., Zheng, F.-J., Lin, B., Wang, T.-S., and Li, Y.-R. (2013). Preparation and characteristics of sugarcane low alcoholic drink by submerged alcoholic fermentation. *Sugar Tech*. 15, 412–416. doi: 10.1007/s12355-013-0248-3”.

The authors apologize for this error and state that this does not change the scientific conclusions of the article in any way. The original article has been updated.

## References

[B1] Chen G.-L. Zheng F.-J. Lin B. Wang T.-S. Li Y.-R. (2013). Preparation and characteristics of sugarcane low alcoholic drink by submerged alcoholic fermentation. Sugar Tech. 15, 412–416. 10.1007/s12355-013-0248-3

[B2] Chen G. L. Zheng F. J. Lin B. Yang Y. X. Fang X. C. Verma K. K.. (2023). Vinegar: a potential source of healthy and functional food with special reference to sugarcane vinegar. Front. Nutr. 10, 1145862. 10.3389/fnut.2023.1145862 37006937 PMC10061008

[B3] Chen G. L. Zheng F. J. Sun J. Li Z. C. Lin B. Li Y. R. (2015). Production and characteristics of high quality vinegar from sugarcane juice. Sugar Tech. 17, 89–93. 10.1007/s12355-014-0352-z

[B4] Yi L. Huang T. Li K. Deng L. Li H. Chen S.. (2017). Development of liquid fermented sugarcane vinegar beverage. Chin. Condiment. 42, 83–88. 10.3969/j.issn.1000-9973.2017.10.01822416693

